# Change of treatment guidelines and evolution of ART initiation in rural South Africa: data of a large HIV care and treatment programme

**DOI:** 10.1186/s12879-015-1207-2

**Published:** 2015-10-26

**Authors:** Mélanie PLAZY, François DABIS, Kevindra NAIDU, Joanna ORNE-GLIEMANN, Till BARNIGHAUSEN, Rosemary DRAY-SPIRA

**Affiliations:** INSERM U897 – Centre Inserm Epidémiologie et Biostatistique, Bordeaux, France; Institut de Santé Publique, d’Epidémiologie et de Développement (ISPED), Université Bordeaux, Bordeaux, France; Africa Centre for Health and Population Studies, University of KwaZulu-Natal, Somkhele, KwaZulu-Natal, South Africa; Department of Global Health and Population, Harvard School of Public Health, Boston, Massachusetts USA; INSERM, UMR_S 1136, Pierre Louis Institute of Epidemiology and Public Health, Team of research in Social Epidemiology, F-75013 Paris, France; Pierre Louis Institute of Epidemiology and Public Health, Team of research in Social Epidemiology, Sorbonne Universités, UPMC Univ Paris 06, UMR_S 1136, F-75013 Paris, France

**Keywords:** South Africa, Antiretroviral therapy, Patient acceptance of health care, Adult, Rural health

## Abstract

**Background:**

While WHO recommendations are to treat people earlier and earlier, it will considerably increase the number of HIV infected people eligible for antiretroviral therapy (ART). In South Africa, a country which carries one of the highest HIV burden worldwide, very few studies are available on the impact of the ART guidelines on time to ART initiation in both individuals with low CD4 count and those newly eligible for ART. We thus aimed to describe ART initiation percentages in a large HIV programme in rural KwaZulu-Natal, South Africa, according to the temporal changes of national ART eligibility guidelines from 2007 to 2012.

**Methods:**

Adults who accessed the decentralized Hlabisa HIV treatment programme in 2007–2012 were included. Three periods following the temporal change of ART eligibility guidelines were defined (Period 1: until April 2010; Period 2: April 2010 - July 2011; Period 3: from August 2011). Percentages of ART initiation within three months of programme entry were estimated in men, in women of childbearing age (<40 years old) and in older women, and stratifying by CD4 count. Trend tests and logistic regression models were used to study the effects of change of guidelines on ART initiation percentages.

**Results:**

In individuals with CD4 count ≤200 cells/μL (*N* = 5709 men, *N* = 6743 women <40 years old and *N* = 2017 older women), percentages of ART initiation did not differ over time (p trend = 0.25; 0.28; and 0.14, respectively). In individuals with CD4 count = 201–350 cells/μL (*N* = 2680 men, *N* = 6086 women <40 years old and *N* = 1415 older women), percentages of ART initiation significantly increased over time (p trend <0.01 for the three groups): from 6 % in Period 1 to 20 % in Period 2 to 40 % in Period 3 in women of childbearing age, and from 7 % to 8-10 % to 42 % in men and in older women.

**Conclusions:**

As temporal changes of guidelines, percentages of ART initiation significantly increased in newly ART eligible people and did not decrease in individuals with very low CD4 counts. It will be crucial to continue verifying the evolution of these percentages of ART initiation with future recommendations reaching near-to-universal access to ART, to ensure that individuals most in need of ART receive it.

## Background

World Health Organization (WHO) guidelines for initiating antiretroviral therapy (ART) have dramatically evolved over the past decade. In 2002, ART were firstly recommended for HIV-infected patients in moderate and advanced stages of infection [1] with the aim to limit disease progression and mortality [2–8]. It was then strongly suggested that ART might be more effective if initiated earlier and in any case before advanced HIV disease [9, 10]. With the evidence of less side-effects, the expansion of ART recommendations was thus suggested in 2006 and reinforced in 2010 for individuals with a CD4 count lower than 350 cells/μL, when most people are still asymptomatic [11, 12]. It was finally shown that ART can lower viral load in HIV-infected individuals [13] and decrease very significantly the risk of HIV transmission within sero-discordant couples [14]. In 2013, WHO guidelines have thus suggested to expand the ART eligibility criteria at CD4 count <500 cells/μL [15]. As WHO recommendations are to treat earlier and earlier in the course of the HIV infection, it is thus critical to inform public health decision-makers of the impact of recommendations changes on time to ART initiation. Specifically, it is important to verify that the time to ART initiation in people with low CD4 count is not longer as the number of newly ART eligible people is increasing with expanded recommendation, and that people who become eligible with higher CD4 count initiate ART quickly even if they do not feel sick [16].

In South Africa, a country which carries one of the highest HIV burden worldwide, with an estimated 6.3 million people who were living with HIV in 2013 with an HIV prevalence equal to 19.1 % in adults aged 15–49 years old [17], recommendations regarding ART eligibility have followed WHO guidelines with some delay [18, 19]. Although ART coverage dramatically increased these last years in South Africa [20], many individuals eligible for ART were not on treatment in 2012 [21]. In order to better prepare the implementation and the consequences of the new WHO recommendations, we thus aimed to describe ART initiation percentages in a large decentralized HIV programme in rural KwaZulu-Natal, South Africa, according to the temporal changes of national ART eligibility guidelines from 2007 to 2012.

## Methods

### Study setting

The decentralized Hlabisa HIV Treatment and Care Programme was initiated in mid-2004 in southern uMkhanyakude district in northern KwaZulu-Natal [22]. This programme takes place in a poor, predominantly rural area, where adult HIV prevalence was about 29 % in 2011, peaking at more than 45 % in women between 25 and 49 year old and almost 30 % in men of the same age [23]. This programme is a partnership between the local Department of Health and the Africa Centre for Health and Population Studies. It is devolved to the 17 primary health care clinics in the sub-district, and is nurse- and counsellor-led. People can access HIV testing and counselling at any time and receive ART for free if they are eligible. Blood samples are taken from people who are diagnosed HIV-positive on rapid test at the clinic and are sent to the local laboratory at Hlabisa district hospital for CD4 testing, with results available at the clinic-level within one week of HIV test. ART eligibility has been assessed according to the SA national guidelines: CD4 count ≤200 cells/μL or WHO stage IV until April 2010; expansion to CD4 count ≤350 cells/μl for people with tuberculosis (TB) and pregnant women from April 2010 [18]; and further expansion to all adults with CD4 count ≤350 cells/μl from August 2011 as per the 2010 WHO guidelines [12]. Before initiating ART, eligible patients attend three ART literacy sessions within a two-week period during which they are also further assessed clinically. Individuals not yet eligible for ART are encouraged to return to the clinic for subsequent monitoring, after one year if CD4 count >500 cells/μL and after six months if CD4 count ≤500 cells/μL. Clinical and laboratory information of both ART-eligible and not-yet ART eligible individuals who accessed one of the 17 primary health care clinics included within this programme are routinely collected and entered in a dedicated database (named ARTemis).

### Available data

Within the ARTemis database, very few data were available for individuals not on ART: information concerning sex and age was collected at the first clinic visit, and CD4 count was collected at each clinic visit. Information on death and transfer was reported over time in clinics; data on death were completed through linkage with the National Population Register performed in October 2011.

### Study population

For this analysis, all adults ≥16 years old who entered the programme between January 1st, 2007 and September 15th, 2012 were included. Entry in the programme was assessed as the date of the first CD4 measure following HIV diagnosis. Individuals were excluded if they were transferred in from outside the Hlabisa programme, if information on sex or first clinic visit was missing, or if the first CD4 count measure was unknown.

### Statistical analysis

Our main outcome was ART initiation within three months of programme entry, according to the cut-off suggested by *Fox et al*. for studying uptake of ART initiation after ART eligibility [24]. Analyses were restricted to participants eligible for ART initiation according to the most recent South African recommendations in our study period, i.e. all individuals with ≤350 cells/μL at programme entry. As pregnancy is an important characteristic for defining ART eligibility (and as the last national antenatal sentinel HIV report [25] suggested that the large majority of women of childbearing age were <40 years old in South Africa) , three groups of individuals were considered: i/ men, ii/ women of childbearing age (less than 40 years old), iii/ older women (40 years or older); these groups were additionally stratified according to baseline CD4 count level (0–200 cells/μL; 201–350 cells/μL). Three entry periods were also defined, categorized according to the changes in ART eligibility criteria over time: period 1 from January 2007 to March 2010, period 2 from April 2010 to July 2011, and period 3 since August 2011. For each of the groups of individuals, and after stratification by CD4 count, percentages of ART initiation within the three months following entry in the programme were globally compared across these three entry periods using trend tests; we then performed univariate logistic regression models for estimating the specific impact of change of guidelines on ART initiation percentages. Individuals who died within the three months following entry in the programme and before ART initiation were excluded. Analyses were carried out using STATA version 11.2 (StataCorp, College Station, Texas).

### Ethics statement

Ethical approval for this analysis of anonymized data from the HIV Treatment and Care Programme was obtained from the University of KwaZulu-Natal (BE066/07) and granted by the Research Office of the KwaZulu-Natal Department of Health, and re-certified annually.

## Results

### Study population

Overall, 44,503 adults entered the HIV Hlabisa programme between January 1, 2007 and September 15, 2012. Among them, 3,552 were excluded from the analysis, of whom 2,544 were patients transferred in from another HIV programme, 730 had a date of first clinic visit before the date of first CD4 count measurement and 15 had a missing clinic name; the others reasons for exclusion were because information on sex was unknown (*N* = 247), and results of the first CD4 measure was unknown (*N* = 16).

Of the remaining 40,951 patients, 24,082 entered the HIV programme in period 1, 8,535 in period 2 and 8,334 in period 3. Among all these individuals, the proportion of women <40 years old remained stable across the three entry periods (56-57 %), the proportion of men increased from 27.6 % during period 1 to 31.0 % during period 3 whereas the proportion of women ≥40 years old decreased from 14.9 % to 12.8 % (*p* < 0.001) (Table 1).

**Table 1:** Description of included individuals according to entry period

	Period 1		Period 2		Period 3		P*
Group–*n* (%)							
*Men*	6,643	(27.6)	2,640	(30.9)	2,582	(31.0)	<0.001
*Women* < *40 years old*	13,851	(57.5)	4,867	(57.0)	4,681	(56.2)	
*Women* ≥*40 years old*	3,588	(14.9)	1,028	(12.1)	1,071	(12.8)	
CD4 count (cells/μL) - median [IQR]							
*In men*	180 [71–341]		183 [74–335]		229 [100–386]		<0.001
*In women <40 years old*	307 [164–481]		288 [158–458]		349 [213–520]		<0.001
*In women ≥40 years old*	258 [135–432]		242 [119–419]		333 [161–522]		<0.001
Age (years)-median [IQR]							
*In men*	36.5 [30.4-44.5]		34.8 [28.7-43.0]		34.5 [28.3-42.0]		<0.001
*In women <40 years old*	27.5 [23.4-32.6]		26.5 [22.5-31.2]		26.7 [22.4-31.6]		<0.001
*In women ≥40 years old*	46.8 [43.2-51.9]		46.9 [42.9-53.0]		48.0 [43.7-53.4]		<0.001

IQR: Inter-Quartile Interval

Period 1: January 1, 2007–March 31, 2010; Period 2: April 1, 2010–July 31, 2011; Period 3: August 1, 2011–September 15, 2012

* *p*.value with Chi2 tests (for group) or Anova tests (for CD4 cells count and Age)

HIV Hlabisa programme, KwaZulu-Natal, South Africa. 2007–2012

Overall, 11,865 men, 23,399 women <40 years old and 5,687 women ≥40 years old accessed the HIV programme across the three entry periods. For these three groups of individuals, baseline median CD4 cell count significantly increased between period 1 and period 3 (*p* < 0.001 for all groups): from 180 cells/μL [Inter-Quartile Range (IQR) 71–341] to 229 cells/μL [IQR 100–386] in men, from 307 cells/μL [IQR 164–481] to 349 cells/μL [IQR 213–520] in women <40 years old and from 258 cells/μL [IQR 135–432] to 333 cells/μL [IQR 161–522] in women ≥40 years old (Table 1). Median age at programme entry varied also according to entry period (*p* < 0.001 for all groups) (Table 1).

### Evolution of ART initiation percentages within three months of entry programme, stratified by group and CD4 count

**Fig. 1:**
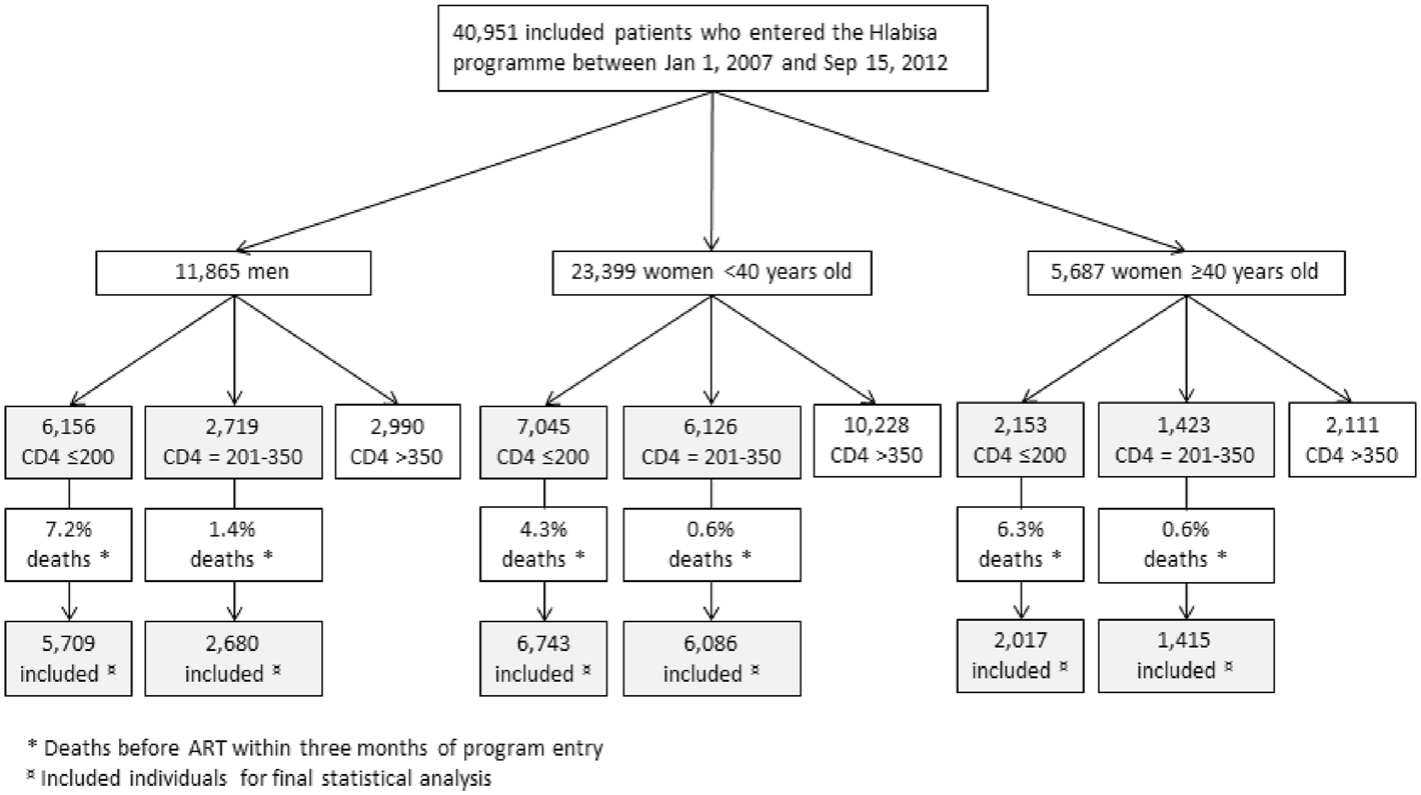
Inclusion of individuals according to sex and CD4 cell count. HIV Hlabisa programme. KwaZulu-Natal, South Africa. 2007–2012

**Fig. 2:**
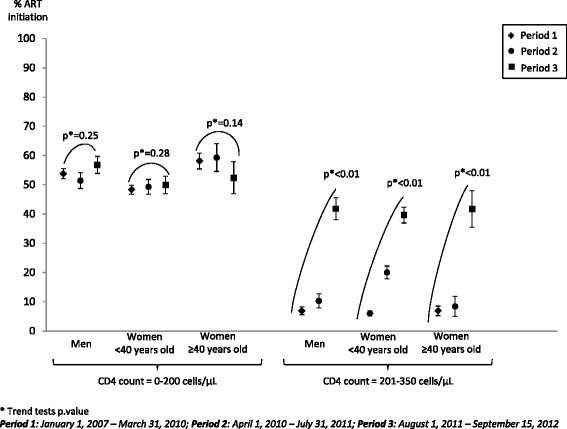
Percentages of ART initiation for each groups (men, women <40 years old, women ≥40 years old), stratified by CD4 count level and entry period. HIV Hlabisa programme. KwaZulu-Natal, South Africa. 2007–2012

In total, 6,156 men, 7,045 women <40 years old and 2,153 women ≥40 years old had a CD4 count ≤200 cells/μL at programme entry. Among them, respectively 7.2 %, 4.3 % and 6.3 % died before ART within three months of programme entry and were thus excluded from the analysis (Fig. 1). Overall percentages of ART initiation within the three months following programme entry among individuals with CD4 count ≤200 cells/μL were 53.8 % [95 % Confidence Interval: 52.6 %-55.2 %] in men (*N* = 5,709), 48.8 % [47.6 %-50.0 %] in women <40 years old (*N* = 6,743) and 57.4 % [55.2 %-59.6 %] in women ≥40 years old (*N* = 2,017). ART initiation percentages varied slightly between period 1 and period 3, from 53.8 % [52.1 %-55.5 %] to 56.8 % [53.9 %-59.6 %] in men, from 48.3 % [46.7 %-49.8 %] to 49.9 % [46.9 %-52.9 %] in women <40 years old, and from 58.1 % [55.3 %-60.7 %] to 52.3 % [46.9 %-57.8 %] in women ≥40 years old (Fig. 2), without significant tendencies according to period entry (p trend = 0.25 in men; p trend = 0.28 in women <40 years old and p trend = 0.14 in women ≥40 years old) (Fig. 2); among these three groups, the odds of ART initiation did not differ between period 2 compared to period 1 or between period 3 compared to period 1 (Table 2).

**Table 2:** ART initiation according to period entry in surviving men, women <40 years old and women ≥40 years old

	Men				Women <40 years old				Women ≥40 years old			
	*N*	ART initiation			*N*	ART initiation			*n*	ART initiation		
		*%*	OR*	*95 % CI*		*%*	OR*	*95 % CI*		*%*	OR*	*95 % CI*
CD4 = 0–200 cells/μL												
Entry period												
Period 1	3 251	53.8	1.00	-	4 136	48.3	1.00	-	1 290	58.1	1.00	-
Period 2	1 324	51.4	0.91	0.80-1.03	1 537	49.3	1.04	0.93-1.17	408	59.3	1.05	0.84-1.32
Period 3	1 134	56.8	1.13	0.98-1.29	1 070	49.9	1.07	0.93-1.22	319	52.3	0.79	0.62-1.01
CD4 = 201–350 cells/μL												
Entry period												
Period 1	1 411	6.9	1.00	-	3 524	6.0	1.00	-	925	6.9	1.00	-
Period 2	611	10.3	1.56	1.12-2.17	1 297	20.0	3.90	3.21-4.73	250	8.4	1.23	0.74-2.06
Period 3	658	41.8	9.73	7.52-12.59	1 265	39.6	10.19	8.53-12.19	240	41.7	9.61	6.70-13.79

Period 1: January 1, 2007–March 31, 2010; Period 2: April 1, 2010–July 31, 2011; Period 3: August 1, 2011–September 15, 2012

OR: Odd-Ratio; 95 % CI: 95 % Confidence Interval

* Logistic regression

HIV Hlabisa Programme, KwaZulu-Natal, South Africa. 2007–2012

In total, 2,719 men, 6,126 women <40 years old and 1,423 women ≥40 years old had a CD4 count between 201 and 350 cells/μL at entry in the programme. Among them, respectively 1.4 %, 0.6 % and 0.6 % died before ART within three months of entry programme and were thus excluded from the analysis (Fig. 1). Overall percentages of ART initiation within three months of programme entry among individuals with CD4 count between 201 and 350 cells/μL were 16.2 % [14.9 %-17.7 %] in men (*N* = 2,680), 16.0 % [15.1 %-16.9 %] in women <40 years old (*N* = 6,086) and 13.1 % [11.4 %-14.9 %] in women ≥40 years old (*N* = 1,415). ART initiation percentages significantly increased over time in the three groups (p trend <0.001 in all the groups, Fig. 2), but at a different pace. In women ≥40 years old and in men, percentages of ART initiation within the three months following programme entry slightly increased between period 1 and period 2 (from 6.9 % [5.5 %-8.7 %] to 8.4 % [5.5 %-12.5 %], Odd-Ratio [OR] 1.23; 95 % Confidence Interval [95 % CI] 0.74-2.06; and from 6.9 % [5.7 %-8.3 %] to 10.3 % [8.1 %-13.0 %], OR 1.56; 95 % CI 1.12-2.17, respectively (Fig. 2, Table 2)); and then dramatically increased to 41.7 % [35.6 %-48.0 %] and 41.8 % [38.1 %-45.6 %], respectively, during period 3 (compared to period 1, OR 9.61; 95 %  CI 6.70-13.79 and OR 9.73; 95 % CI 7.52-12.59, respectively (Fig. 2, Table 2)). In women <40 years old, percentages of ART initiation within the three months following programme entry showed a marked increase between period 1 and period 2, from 6.0 % [5.3 %-6.9 %] to 20.0 % [18.0 %-22.3 %] (OR 3.90; 95 % CI 3.21-4.73); and then further increased to 39.6 % [36.9 %-42.3 %] during period 3 (compared to period 1, OR 10.19; 95 % CI 8.53-12.19) (Fig. 2, Table 2).

## Discussion

In this rural area of South Africa, as guidelines regarding ART eligibility criteria have been expanded, percentages of ART initiation within three months of programme entry have clearly increased for individuals eligible for ART according to new criteria (CD4 count equal to 201–350 cells/μL), without decreasing for individuals with very low CD4 count (CD4 count lower than 200 cells/μL). Evolution of ART initiation percentages matched the expansion of ART eligibility guidelines in individuals with a baseline CD4 count comprised between 201 and 350 cells/μL. In women of childbearing age, ART initiation percentages significantly gradually increased during the three study periods. In men and in women ≥40 years old, a slight increase was observed between the first and the second period (when ART eligibility criteria was also expanded to TB co-infected individuals with a CD4 count ≤350 cells/μL), but this increase in ART initiation percentages was most important between the second and the third entry period (when all individuals with a CD4 count ≤350 cells/μL became eligible for ART). These results remained unchanged when adjusted for age (data not shown).

A previous modelling study showed that the number of people eligible for ART in South Africa increased from 1.7 million to 2.6 million after the change of ART eligibility criteria from CD4 ≤ 200 cells/μL to CD4 ≤ 350 cells/μL [26]. Our results from a rural South African study area suggest that the HIV programme, with the funding available at this time (PEPFAR/USAID and the KZN Department of Health), could adapt on the field to the change of national guidelines, with a number of individuals accessing clinics for initiating ART that increased over time and with higher CD4 count at the date of ART initiation as showed in a previous analysis [27].

It has then been estimated that expanding the ART eligibility criteria to CD4 ≤ 500 cells/μL would dramatically increase the number of individuals eligible for ART to 4.1 million countrywide [26]. Thus, the true test of the impact of the change of guidelines will be with the evaluation of the implementation of the 2013 WHO guideline that has begun in January 2015 in South Africa (http://www.iol.co.za/news/politics/hiv-drugs-treatment-to-start-earlier-motsoaledi-1.1724412#.VDuOLRbH-ZT), and especially within the Hlabisa sub-district where the HIV care and treatment programme is, since 2013, only funded by the KwaZulu-Natal Department of Health.

In spite of the positive trends in ART initiation, the proportion of eligible individuals who initiated ART remained far from optimal. Indeed, during entry period 3, and for both men and women, percentages of ART initiation within three months of programme entry were <60 % in individuals with a CD4 count ≤200 cells/μL and <50 % in those with a CD4 count between 201 and 350 cells/μL. This is consistent with previous studies conducted in this setting [28] as well as in other sub-Saharan African contexts [29–31] that showed that ART initiation percentages significantly decreased with increasing CD4 cell counts; some authors suggest that the perception of being or feeling sick can be associated with ART initiation [32, 33]. While WHO recommendations aim to treat more people earlier in the course of the HIV infection [15], these results observed in a region of high HIV prevalence suggest that it will be crucial to develop interventions to further increase ART initiation percentages in all ART-eligible patients. It has been suggested that involvement of community health workers may be helpful for increasing ART scale up among individuals eligible while they are still asymptomatic [34]. It has also been shown that the development of the CD4 point-of-care strategy – that reduces the numbers of visits in clinics–could increase retention of patients in HIV care and ART initiation percentages [35–37]. Finally, home-based ART initiation seems to be effective for increasing ART initiation percentages [38]; a study also suggested that the home-based HIV and ART care strategy is not associated with negative patient outcomes [39].

Although this study is based on data of a large HIV treatment and care programme, some individuals may have initiated ART outside the programme, which may have led to an underestimation of the ART initiation percentages. However, this bias is likely to be limited since the Hlabisa HIV programme is decentralized in primary health care clinics with relatively easy access and this area is rural and poor, making it difficult for people to access ART somewhere else. Another limitation is that among included participants, some people may have failed to return to the clinic to receive their CD4 count result after HIV testing and thus were unaware of their status regarding ART eligibility [40–44]; however we cannot provide a precise figure as this information was not collected in the database. Moreover, we were able to update in October 2011 the mortality figure through linkage with the National Population register. Mortality estimates are thus probably more robust in Periods 1 and 2 compared to Period 3; as death was an exclusion criteria when estimating percentages of ART initiation, it is possible that these percentages were underestimated in Period 3. Lastly, WHO stage and pregnancy were not available in the database for people not on ART; we only considered sex and age for defining the three groups of individuals on which we described the evolution of ART initiation percentages.

## Conclusion

In conclusion, the percentages of ART initiation significantly increased in individuals newly eligible for ART after ART eligibility criteria expansion, without decreasing in individuals with advanced disease. However, these percentages of ART initiation remained low three months after programme entry. While future recommendations aim at reaching near-to-universal access to ART, it will be crucial to continue verifying the evolution of ART initiation percentages according to these new recommendations, as well as intensifying efforts in order to rapidly maximize ART initiation after accessing an HIV programme in all individuals eligible for ART, whether they are at an advanced stage of HIV infection or not.

## References

[1] WHO. Scaling up antiretroviral therapy in resource-limited settings. 2002 [Cited 09/09/2015]. Available from: http://www.who.int/hiv/pub/prev_care/en/ScalingUp_E.pdf.

[2] Bor J, Herbst AJ, Newell ML, Barnighausen T (2013). Increases in adult life expectancy in rural South Africa: valuing the scale-up of HIV treatment. Science.

[3] Gargano JW, Laserson K, Muttai H, Odhiambo F, Orimba V, Adamu-Zeh M (2012). The adult population impact of HIV care and antiretroviral therapy in a resource poor setting, 2003–2008. AIDS.

[4] Herbst AJ, Cooke GS, Barnighausen T, KanyKany A, Tanser F, Newell ML (2009). Adult mortality and antiretroviral treatment roll-out in rural KwaZulu-Natal, South Africa. Bull World Health Organ.

[5] Hermans SM, Van Leth F, Manabe YC, Hoepelman AI, Lange JM, Kambugu A (2012). Earlier initiation of antiretroviral therapy, increased tuberculosis case finding and reduced mortality in a setting of improved HIV care: a retrospective cohort study. HIV Med.

[6] Iwuji CC, Mayanja BN, Weiss HA, Atuhumuza E, Hughes P, Maher D (2011). Morbidity in HIV-1-infected individuals before and after the introduction of antiretroviral therapy: a longitudinal study of a population-based cohort in Uganda. HIV Med.

[7] Kasamba I, Baisley K, Mayanja BN, Maher D, Grosskurth H (2012). The impact of antiretroviral treatment on mortality trends of HIV-positive adults in rural Uganda: a longitudinal population-based study, 1999–2009. Trop Med Int Health.

[8] Moh R, Danel C, Messou E, Ouassa T, Gabillard D, Anzian A (2007). Incidence and determinants of mortality and morbidity following early antiretroviral therapy initiation in HIV-infected adults in West Africa. AIDS.

[9] Walensky RP, Wolf LL, Wood R, Fofana MO, Freedberg KA, Martinson NA (2009). When to start antiretroviral therapy in resource-limited settings. Ann Intern Med.

[10] Grinsztejn B, Hosseinipour MC, Ribaudo HJ, Swindells S, Eron J, Chen YQ (2014). Effects of early versus delayed initiation of antiretroviral treatment on clinical outcomes of HIV-1 infection: results from the phase 3 HPTN 052 randomised controlled trial. Lancet Infect Dis.

[11] WHO. Antiretroviral therapy for HIV infection in adults and adolescents: recommendations for a public health approach. 2006 (revision) [Cited 09/09/2015]. Available from: http://www.who.int/hiv/pub/guidelines/artadultguidelines.pdf?ua=1. 23741771

[12] WHO. Antiretroviral therapy for HIV infection in adults and adolescents. Recommandations for a public health approach. 2010 (revision) [Cited Available from: http://whqlibdoc.who.int/publications/2010/9789241599764_eng.pdf. Access date: 09/09/2015] 23741771

[13] Attia S, Egger M, Muller M, Zwahlen M, Low N (2009). Sexual transmission of HIV according to viral load and antiretroviral therapy: systematic review and meta-analysis. AIDS.

[14] Cohen MS, Chen YQ, McCauley M, Gamble T, Hosseinipour MC, Kumarasamy N (2011). Prevention of HIV-1 infection with early antiretroviral therapy. N Engl J Med.

[15] WHO. Consolidated guidelines on the use of antiretroviral drugs for treating and preventing HIV infection: Recommendations for a public health approach. 2013 [Cited 09/09/2015]. Available from: http://www.who.int/iris/bitstream/10665/85321/1/9789241505727_eng.pdf. 24716260

[16] Eholie SP, Vella S, Anglaret X (2014). Commentary: Antiretroviral therapy initiation criteria in low resource settings--from ‘when to start’ to ‘when not to start’. AIDS.

[17] UNAIDS. The gap report. 2014 [Cited 09/09/2015]. Available from: http://www.unaids.org/sites/default/files/media_asset/UNAIDS_Gap_report_en.pdf.

[18] Departement of Health of the Republic of South Africa. The South African antiretroviral treatment guidelines 2010. 2010 [Cited 09/09/2015]. Available from: http://www.uj.ac.za/en/corporateservices/ioha/documentation/documents/art%20guideline.pdf.

[19] Departement of Health of the Republic of South Africa. The South African antiretroviral treatment guidelines 2013. 2013 [Cited 09/09/2015]. Available from: http://www.sahivsoc.org/upload/documents/2013%20ART%20Guidelines-Short%20Combined%20FINAL%20draft%20guidelines%2014%20March%202013.pdf.

[20] WHO, UNICEF, UNAIDS. Global update on HIV treatment 2013: Results, impact and opportunities. 2013 [Cited 09/09/2015]. Available from: http://www.who.int/hiv/pub/progressreports/update2013/en/index.html.

[21] UNAIDS. Global report: UNAIDS report on the global AIDS epidemic. 2013 [Cited 09/09/2015]. Available from: http://www.unaids.org/en/media/unaids/contentassets/documents/epidemiology/2013/gr2013/UNAIDS_Global_Report_2013_en.pdf.

[22] Houlihan CF, Bland RM, Mutevedzi PC, Lessells RJ, Ndirangu J, Thulare H (2011). Cohort profile: Hlabisa HIV treatment and care programme. Int J Epidemiol.

[23] Zaidi J, Grapsa E, Tanser F, Newell ML, Barnighausen T (2013). Dramatic increase in HIV prevalence after scale-up of antiretroviral treatment. AIDS.

[24] Fox MP, Larson B, Rosen S (2012). Defining retention and attrition in pre-antiretroviral HIV care: proposals based on experience in Africa. Trop Med Int Health.

[25] Departement of Health of the Republic of South Africa. The 2012 National Antenatal Sentinel HIV and Herpes Simplex type-2 prevalence survey, South Africa. 2012 [Cited 09/09/2015]. Available from: http://www.health-e.org.za/wp-content/uploads/2014/05/ASHIVHerp_Report2014_22May2014.pdf.

[26] Hontelez JA, Newell ML, Bland RM, Munnelly K, Lessells RJ, Barnighausen T (2012). Human resources needs for universal access to antiretroviral therapy in South Africa: a time and motion study. Hum Resour Health.

[27] Lessells RJ, Mutevedzi PC, Iwuji CC, Newell ML (2014). Reduction in early mortality on antiretroviral therapy for adults in rural South Africa since change in CD4+ cell count eligibility criteria. J Acquir Immune Defic Syndr.

[28] Plazy M, Dray-Spira R, Orne-Gliemann J, Dabis F, Newell ML (2014). Continuum in HIV care from entry to ART initiation in rural KwaZulu-Natal, South Africa. Trop Med Int Health.

[29] Geng EH, Bwana MB, Muyindike W, Glidden DV, Bangsberg DR, Neilands TB (2013). Failure to initiate antiretroviral therapy, loss to follow-up and mortality among HIV-infected patients during the pre-ART period in Uganda. J Acquir Immune Defic Syndr.

[30] Aliyu MH, Blevins M, Parrish DD, Megazzini KM, Gebi UI, Muhammad MY (2014). Risk factors for delayed initiation of combination antiretroviral therapy in rural north central Nigeria. J Acquir Immune Defic Syndr.

[31] Parkes-Ratanshi R, Bufumbo L, Nyanzi-Wakholi B, Levin J, Grosskurth H, Lalloo DG (2010). Barriers to starting ART and how they can be overcome: individual and operational factors associated with early and late start of treatment. Trop Med Int Health.

[32] Fox MP, Mazimba A, Seidenberg P, Crooks D, Sikateyo B, Rosen S (2010). Barriers to initiation of antiretroviral treatment in rural and urban areas of Zambia: a cross-sectional study of cost, stigma, and perceptions about ART. J Int AIDS Soc.

[33] Duff P, Kipp W, Wild TC, Rubaale T, Okech-Ojony J (2010). Barriers to accessing highly active antiretroviral therapy by HIV-positive women attending an antenatal clinic in a regional hospital in western Uganda. J Int AIDS Soc.

[34] Hermann K, Van Damme W, Pariyo GW, Schouten E, Assefa Y, Cirera A (2009). Community health workers for ART in sub-Saharan Africa: learning from experience--capitalizing on new opportunities. Hum Resour Health.

[35] Faal M, Naidoo N, Glencross DK, Venter WD, Osih R (2011). Providing immediate CD4 count results at HIV testing improves ART initiation. J Acquir Immune Defic Syndr.

[36] Jani IV, Sitoe NE, Alfai ER, Chongo PL, Quevedo JI, Rocha BM (2011). Effect of point-of-care CD4 cell count tests on retention of patients and rates of antiretroviral therapy initiation in primary health clinics: an observational cohort study. Lancet.

[37] Larson BA, Schnippel K, Ndibongo B, Xulu T, Brennan A, Long L (2012). Rapid point-of-care CD4 testing at mobile HIV testing sites to increase linkage to care: an evaluation of a pilot program in South Africa. J Acquir Immune Defic Syndr.

[38] MacPherson P, Lalloo DG, Webb EL, Maheswaran H, Choko AT, Makombe SD (2014). Effect of optional home initiation of HIV care following HIV self-testing on antiretroviral therapy initiation among adults in Malawi: a randomized clinical trial. JAMA.

[39] Apondi R, Bunnell R, Awor A, Wamai N, Bikaako-Kajura W, Solberg P (2007). Home-based antiretroviral care is associated with positive social outcomes in a prospective cohort in Uganda. J Acquir Immune Defic Syndr.

[40] Clouse K, Pettifor AE, Maskew M, Bassett J, Van Rie A, Behets F (2013). Patient retention from HIV diagnosis through one year on antiretroviral therapy at a primary health care clinic in Johannesburg, South Africa. J Acquir Immune Defic Syndr.

[41] Govindasamy D, Van Schaik N, Kranzer K, Wood R, Mathews C, Bekker LG (2011). Linkage to HIV care from a mobile testing unit in South Africa by different CD4 count strata. J Acquir Immune Defic Syndr.

[42] Larson BA, Brennan A, McNamara L, Long L, Rosen S, Sanne I (2010). Lost opportunities to complete CD4+ lymphocyte testing among patients who tested positive for HIV in South Africa. Bull World Health Organ.

[43] Amuron B, Namara G, Birungi J, Nabiryo C, Levin J, Grosskurth H (2009). Mortality and loss-to-follow-up during the pre-treatment period in an antiretroviral therapy programme under normal health service conditions in Uganda. BMC Public Health.

[44] Losina E, Bassett IV, Giddy J, Chetty S, Regan S, Walensky RP (2010). The “ART” of linkage: pre-treatment loss to care after HIV diagnosis at two PEPFAR sites in Durban, South Africa. PLoS One.

